# Comparison of invasive histological and molecular methods in the diagnosis of *Helicobacter pylori* from gastric biopsies of Sudanese patients: a cross-sectional study

**DOI:** 10.12688/f1000research.75873.2

**Published:** 2022-06-08

**Authors:** Maram Elnosh, Hisham Altayb, Yousif Hamedelnil, Wafa Elshareef, Aliaa Abugrain, Esraa Osman, Aalaa Albasha, Abdelhamid Abdelhamid, Ehssan Moglad, Ahmed AbdAlla, Ahmed Ismail

**Affiliations:** 1 Microbiology, College of Medical Laboratory Science, Sudan University of Science and Technology, Khartoum, State, 11111, Sudan; 2 Biochemistry, Faculty of Sciences, King Abdulaziz University, Jeddah, State, 21452, Saudi Arabia; 3 Histopathology, The National Public Health Laboratory, Khartoum, State, 11111, Sudan; 4 Histopathology, College of Medical Laboratory Science, Sudan University of Science and Technology, Khartoum, State, 11111, Sudan; 5 Pharmaceutics, College of Pharmacy, Prince Sattam bin Abdulaziz University, Alkharj, State, 11942, Saudi Arabia; 6 Parasitology and Medical Entomology, College of Medical Laboratory Science, Sudan University of Science and Technology, Khartoum, State, 11111, Sudan; 7 Public Health Department, Ministry of Public Health, Doha, State, 122104, Qatar

**Keywords:** Helicobacter pylori, Histopathology, 16S rRNA, PCR, ureA, sensitivity, specificity, Khartoum.

## Abstract

**Background: **The continuous rise in the number of patients suffering from *Helicobacter pylori* is probably due to the changes in modern life. Nowadays, patients suffering from gastrointestinal problems are diagnosed through invasive and non-invasive techniques. The choice of a diagnostic test is influenced by factors such as the tests' sensitivity and specificity, the clinical conditions, and the cost-effectiveness of the testing strategy. This study aimed to compare molecular detection methods of *H. pylori* by polymerase chain reaction (PCR) targeting the *16S rRNA, ureA *and *glmM* genes with an invasive histopathological technique.

**Methods: **290 gastric biopsies were collected using gastrointestinal endoscopy from patients with gastritis symptoms in different hospitals in Khartoum state. Two gastric biopsies were collected from each patient for PCR and histopathology.

**Results**: A total of 103 (35.5%) samples were positive by histopathological examination, 88 (30.3%) by *16S rRNA*, 39 (13.4%) by *glmM* gene, and 56 (19.3%) by *ureA* gene. The highest sensitivity was observed in *16S rRNA *(46.6%), followed by *glmM* (24.3%) and *ureA* (23.3%). While the best specificity was observed in *glmM* gene (92.5%), followed by *ureA* (82.3%) and *16S rRNA* (78.6%).

**Conclusion**: PCR test targeting the *16S rRNA* gene exhibited the best results for molecular detection of *H. pylori *compared to other genes.

## Introduction

*Helicobacter pylori* ( *H. pylori*) is a Gram-negative, microaerophilic, spiral, and motile bacterium that colonizes the human gastric mucosa. ^1^^,^^2^ It has been associated with the development of various clinical disorders of the upper gastrointestinal tract, such as aseptic ulcers, chronic gastritis, gastric adenocarcinoma and gastric mucosa-associated lymphoid tissue (MALT) lymphoma, which is classified as type I cancer-causing agent by the World Health Organization (WHO). ^3^^–^^5^ Its distribution is worldwide and affects more than 90% of the world population, but it is more common in developing countries with the highest prevalence found in Africa, ^6^^,^^7^ probably due to the possible transmission through the fecal-oral route and the unsafe sanitation conditions in these countries. ^1^^,^^8^ Clinically, a variety of various invasive techniques (requiring endoscopy and biopsy which include, culture, histological examination, and rapid urease test, CLO (Campylobacter like organism) test, smear examination, and molecular studies such as polymerase chain reaction (PCR) and fluorescence *in situ* hybridization (FISH)) or noninvasive techniques (including serology, respiratory urea breath test, or the detection of fecal antigen) are often performed to detect *H. pylori* infection. FISH with *16S rRNA* oligonucleotide probes has been used for detection and identification of *H. pylori* and detection of resistance to antimicrobials ^9^^–^^11^ The sensitivity of any of those techniques in detecting *H. pylori* relays on how the density of the bacterial cells within the specimens taken by biopsy (recent use of disease-related medications, specifically antibiotics and proton-pump inhibitors (PPI) can reduce the density of the cells), pathologist expertise, also the type and quality of the stain used for detection purposes. ^10^ Many studies reported that the gold standard method for the diagnosis is the detection of *H. pylori* in biopsy material. ^12^^,^^13^

Currently, many clinical laboratories use urease tests and histological analysis as a gold standard approach. ^13^^,^^14^ In routine practice, hematoxylin and eosin (H and E), Giemsa, and immunohistochemistry staining techniques are commonly used to identify *H. pylori* following endoscopy; however, these techniques normally fails in identifying low numbers or coccoid forms of bacteria. ^15^

The polymerase Chain Reaction (PCR) method offers advantages over culture and histopathology because it can detect the coccoid form of the *H. pylori.* PCR which is highly specific and sensitive for the diagnosis of *H. pylori* from gastric biopsy, saliva, urine and stool specimen, as well as for detection of virulence and drug resistance genes especially clarithromycin resistance. ^16^ The targets of these PCR methods include the *16S rRNA* gene, the urease ( *ureA*) gene, the *ureC* gene, renamed phosphoglucosaminemutase ( *glmM*), the random chromosome sequence, and the 26-kDa species-specific antigen (SSA) gene. *H. pylori ureA* gene is an important virulence factor that ensures that bacteria can resist acidity of the gastric mucosa. ^17^

In Sudan, many studies were carried out to investigate the seroprevalence of *H. pylori* using ELISA and rapid immunochromatographic tests. ^18^ The prevalence of *H. pylori* infection was estimated to be 80% among patients with gastritis symptoms, 56% with duodenal ulcer, while 60% with duodenitis and 16% apparently healthy individuals. ^19^ In another study in Eastern Sudan high prevalence of *H. pylori* infection, 80% among patients with gastritis and Barrett's esophagus was reported. ^20^ In Sudan and probably many third-world countries, the cost of diagnosis plays a major role rather than the accuracy of the diagnostic method. Hence, diagnosis of *H. pylori* infections is largely based on serology, detection of stool antigen and rarely endoscopy and culture. The present study aimed to compare the use of histopathology (gold standard method) with polymerase chain reaction (PCR) approach for the detection and prevalence of *H. pylori* infections in Khartoum State.

## Methods

This was a cross-sectional study conducted at Khartoum State, Sudan between March 2018 to January 2020. The project was approved by the Ethics Committee of the Ministry of Health Research Department, Khartoum State (3/2018). The study aims were explained to the recruits, and a consent form was obtained and signed prior to sample collection.

### Collection of biopsy specimens

Out of 290 male and female patients from all age groups who subjected for gastric biopsy through Oesophago-Gastro-Duodenoscopy (OGD) by physicians in different hospitals at Khartoum State (Khartoum locality and Omdurman locality) and suffering from dyspepsia and other gastritis-related symptoms were enrolled in this study in period between March 2018 to January 2020. Patients who had received antibiotics, PPI, H2 blockers, or colloidal bismuth sulfate within the previous two months of endoscopy for treatment of gastritis or peptic ulcer, patients with a history of gastric resection, patients with complicated peptic ulcer disease, *i.e.* hemorrhage, were excluded. ^4^ Two biopsy specimens were collected from the antrum and the corpus of each patient, one sample was immediately placed in tubes containing saline and transported for molecular study, while the other was fixed in 10% buffered formalin for at least 24 hours and then embedded in paraffin wax for histopathological examination.

### Histopathological identification of *Helicobacter pylori*

Hematoxylin and Eosin (H and E) staining and modified Giemsa staining were performed for all samples. Three sections for each specimen were deparaffinized and hydrated in descending grades of alcohol and cut in sequential 4 μm sections. One slide was stained by routine H and E stain, and the other slide was stained by modified Giemsa stain to demonstrate the presence of *H. pylori.* Cover slips with DPX mounted on slides and then later examined by a histopathologist and assigned to each morphological variable.

### DNA extraction

DNA extraction of gastric biopsies was performed using the guanidine chloride method as described by Abd Al Rahem and Elhag. ^21^ Biopsies were grounded by sterile blades and tips and then washed with phosphate buffer saline (PBS). 2 ml of lysis buffer were added, followed by 10 μl of proteinase K, 1 ml of guanidine chloride, and 300 μl of ammonium (NH _4_) acetate, then vortexed and incubated at 65°C for 2 hours. The mixture was cooled to room temperature, and then 2 ml of pre-cooled chloroform was applied, vortexed, and centrifuged for 5 minutes at 3000 revolutions per minute (rpm). The upper layer of the mixture was moved to a new tube, and 10 ml of absolute cold ethanol were added, shaken, and held for 2 hours or overnight at −20°C. The tube was then centrifuged for 15–20 minutes at 3000 rpm, the supernatant was carefully removed, and the tube was inverted for 5 minutes on tissue paper. The pellet was washed with 70% ethanol, centrifuged for 5 minutes at 3000 rpm. The supernatant was poured away, allowing the pellet to dry for 10 minutes. Then re-suspended into 50 μl of distilled water, briefly vortexed, and held overnight at −20°C. The extracted DNA was stored at −80°C until use.

### Polymerase chain reaction (PCR)

Three different primers were used for the detection of the bacteria, targeting specific *H. pylori 16S rRNA* (532 bp), *glmM* (294 bp), and *ureA* (217 bp). PCR was carried out in 25 μl of reaction mixture containing 5 μl of ready to use master mix (Taq DNA polymerase, dNTPs and MgCl _2_) (Intron Biotechnology, Korea), 2 μl of DNA template, 1 μl of forward (F) primer, 1 μl of reverse (R) primer and 16μl distilled water (DW). For each batch of PCR assay, DW was used as negative control instead of the genomic DNA templates and known positive sample was used as positive control. The reaction mixtures were cycled in an automated thermocycler. The PCR for the specific *H. pylori 16S rRNA* gene was performed using the forward primer (5′-GCTAAGAGATCAGCCTATGTCC-3′) and reverse primer (5′-TGGCAATCAGCGTCAGGTAAT-3′). The PCR condition for the *16S rRNA* gene was performed as follows: initial denaturation at 94°C for 3 minutes, 35 cycles of denaturation at 94°C for 30 seconds, annealing at 53°C for 30 seconds, extension at 72°C for 45 seconds, and a final extension at 72°C for 5 minutes. ^22^ The PCR for the *ureA* gene of *H. pylori* was performed using the forward primer (5′-AACCGGATGATGTGATGGAT-3′) and reverse primer (5′-GGTCTGTCGCCAACATTTTT-3′) reported by Ye *et al*., which results in an amplicon of 217 bp. The PCR condition for the *ureA* gene was performed as follows: initial denaturation at 94°C for 3 minutes, 35 cycles of denaturation at 94°C for 30 seconds, annealing at 53°C for 30 seconds, extension at 72°C for 45 seconds, and a final extension at 72°C for 5 minutes. ^22^

The PCR for the *glmM* gene was performed using the forward primer (5′-GGATAAGCTTTTAGGGGTGTTAGGGG-3′) and reverse primer (5′-GCTTACTTTCTAACACTAACGCGC-3′). ^23^ The PCR condition for the *glmM* gene was performed as follows: initial denaturation at 94°C for 3 minutes, 35 cycles of denaturation at 94°C for 30 seconds, annealing at 58°C for 30 seconds, extension at 72°C for 30 seconds, and a final extension at 72°C for 3 minutes.

After amplification, 5 μl of the product was run in electrophoreses on a 1.5% agarose gel containing Ethidium bromide (0.5 μg/ml), then visualized under an ultraviolet illuminator and photographed. A 100-bp DNA ladder was used as a size marker (Intron Biotechnology, Korea).

### Statistical analysis

Statistical analysis was done using IBM Statistical Package for Social Sciences (SPSS) software version 20.0 (RRID: SCR_019096 URL: https://www.ibm.com/products/spss-statistics). Chi-squared test was done for the analysis of categorical variables. A *p*-value of <0.05 was considered statistically significant.

## Results

The sociodemographic and clinical data of 290 patients recruited in this study are shown in Table 1.

**Table 1.** Sociodemographic and clinical data for participated patients.

Patients characters (n=290)	Number (%)
**Sex**	
Males	159 (54.8)
Females	131 (45.1)
**Age (years)**	
14-29	72 (24.8%)
30-49	117 (40.3%)
50 years and older	101 (34.8%)
**Residence** *	
Khartoum locality	175 (60.3)
Omdurman locality	115 (39.7)
**Endoscopy**	
gastritis	194 (66.9)
gastric ulcer	29 (10.0)
duodenal ulcer	27 (9.3)
esophagitis	13 (4.5)
normal gastric mucosa	27 (9.3)
**Signs and symptoms**	
Dyspepsia	118 (40.7)
Vomiting	26 (9.0)
Dysphagia	22 (7.6)
Abdominal pain	72 (24.8)
Acidity	52 (17.9)

* Samples were collected from two localities in Khartoum State.

### Histopathological identification of *Helicobacter pylori*

Gastric biopsies were obtained from 290 patients suffering from various gastric conditions through Oesophago-Gastro-Duodenoscopy (OGD). *H. pylori* were clearly detected in positive samples as curved bacilli on the surface of the gastric epithelial cells; the bacteria appear as light bluish rods in H and E slides with varying sizes (3–6 μ) on the luminal surface of mucosal cells. In Giemsa’s stain *H. pylori* appear dark blue in a light blue background. ^3^

From a total of 290 samples, *H. pylori* were found in 103 samples (35.5%). The highest number of positive *H. pylori* samples were observed in the active chronic gastritis followed by patients of the duodenal ulcer, gastric ulcer, and normal gastric findings in the following frequencies: 75 (25.9%), 13 (4.5%), 6 (2.1%) and 6 (2.1%) respectively, while the lowest frequency was noticed in patients with esophagitis 3 (1.0%) cases.

Patients enrolled in the study were divided into three age groups: young adults 14-29 years, middle-aged adults 30-49 years, and old-aged adults 50 years and older. The detection of *H. pylori* infection was 27 (9.3%), 45 (15.5%), and 31 (10.7%), respectively. The prevalence of *H. pylori* increased gradually with age, but it was statistically insignificant ( *p* = 0.451).

### Detection of *H. pylori* 16S rRNA, glmM, and ureA genes of *H. pylori* by PCR

**Figure 1.**
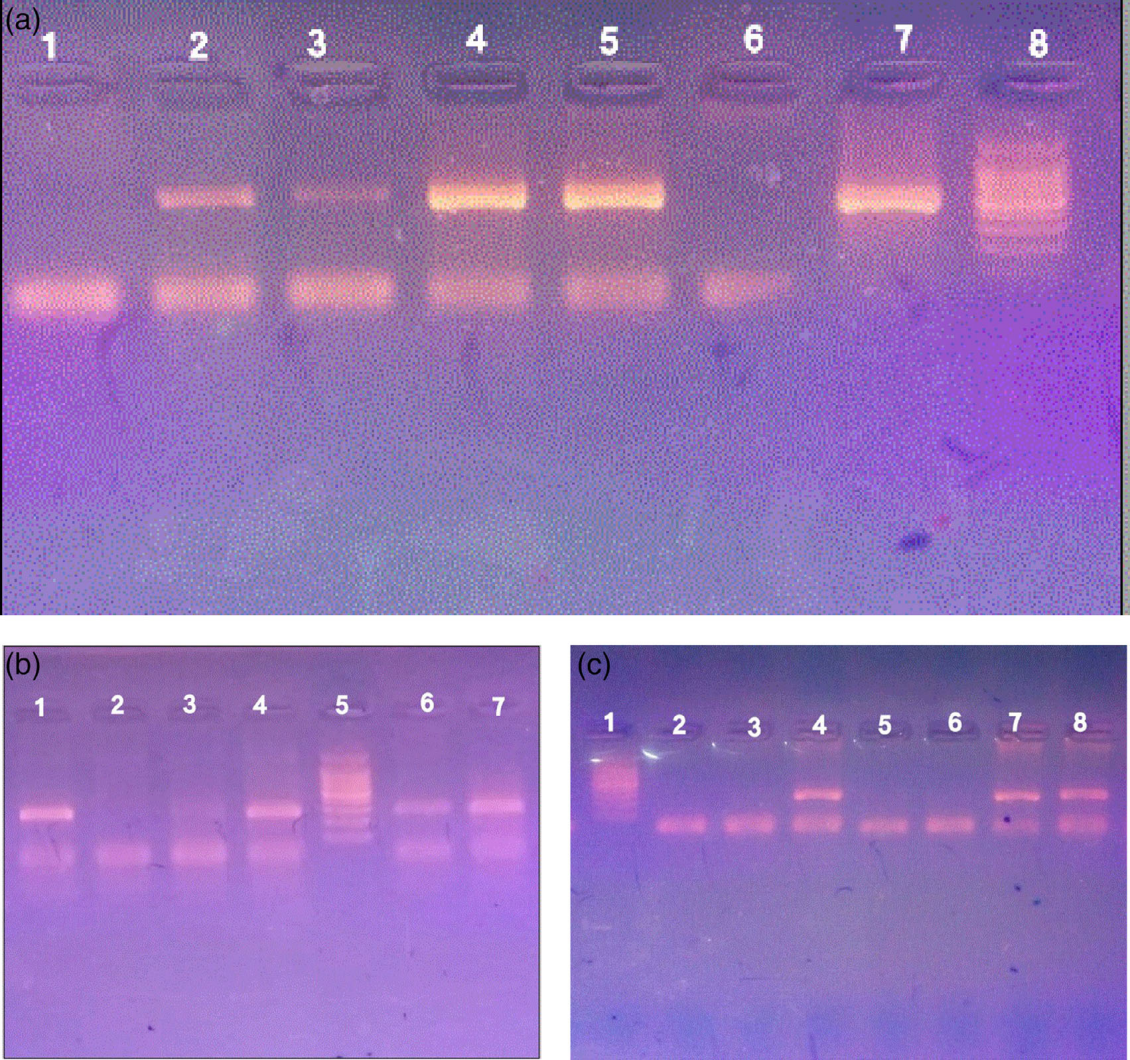
PCR amplification of *H. pylori* on agarose gel electrophoresis 1.5%. a. *16S rRNA* gene. Lane 8 marker (100–1500 bp), lane 7 positive control, lanes 2–5 contain positive samples (532 bp), lanes 1 and 6 are negative samples. b. *glmM* gene, lane 5 marker (100–1500 bp), lane 4 positive control, lanes 1,6, and 7 contain amplicons of *glmM* (294 bp), lanes 2 and 3 are negative samples. c. *ureA* gene. Lane 1 marker (100–1500 bp), lane 8 positive control, lanes 4 and 7 contain amplicons of *ureA* (217 bp), lanes 2, 3, 5, and 6 are negative samples.

Among the samples analyzed by the PCR method for *H. pylori* 88 (30.3%) were positive using *H. pylori* *16S rRNA* gene, 39 (13.4%) samples were positive using *glmM* gene, 56 (19.3%) samples were positive using *ureA* gene, and the rest of samples 234 (80.7%) were negative ( Figure 1).

Considering the histology as a gold standard, the PCR method using *16S rRNA* were the most sensitive methods (46.6%). The PCR method using *glmM* gene were the most specific method (92.5%). The PPV, NPV and odds ratio of each method are noted in Table 2.

**Table 2.** Comparison between histopathological approach and various PCR methods used for the diagnosis of *H. pylori* infections in this study.

PCR methods		Histopathological technique		*p*-value	Odds ratio	Sensitivity (%)	Specificity (%)	PPV (%)	NPV (%)
		Positive	Negative						
*16S rRNA*	**Positive**	48	40	<0.05	3.207	46.6	78.6	54.5	72.8
	**Negative**	55	147						
*glmM*	**Positive**	25	14	<0.05	3.961	24.3	92.5	64.1	68.9
	**Negative**	78	173						
*ureA*	**Positive**	24	32	0.201	1.472	23.3	82.3	42.9	66.2
	**Negative**	79	155						
Total		103	187						

## Discussion

Currently, there are many diagnostic methods for the diagnosis of *H. pylori* infections; each method has its advantages and disadvantages, so it is recommended to use at least a combination of two methods based on different principles to detect colonization by *H. pylori.* ^24^ Although, the culture method is regarded as the most appropriate technique, it has limitations, particularly in case of slow-growing or fastidious bacteria, due to complicated identification and time-consuming methods. In addition to the need for immediate transport of the biopsy specimens to the designated laboratory to assure the viability of *H. pylori* and prevent the formation of coccoid forms of the microorganism. ^24^^–^^26^ The histological technique and culturing of gastric biopsy specimens have been considered a gold standard method under optimal conditions. ^24^

Histological staining enables identifying bacteria and evaluating the type and intensity of the gastric mucosa's inflammation and associated pathology, such as, atrophic gastritis (AG), intestinal metaplasia (IM), and gastric cancer or lymphoma. ^27^

In this study, the prevalence of *H. pylori* infection was 35.5%. *H. pylori* was detected in (103/290) patients using histopathological examination with 35.5% sensitivity. There are many previous studies done in this field with various pictures of the disease. Mohamed *et al.* reported that 16/69 (23.2%) positive patients for *H. pylori* infection among Sudanese patients with colon polyps and colon cancer patients. ^18^ Redéen *et al.* reported that 97/304 (31.9%) positive patients for *H. pylori* infection. ^28^ In another study, Salman *et al.* reported that 115/210 (54.7%) samples were positive for *H. pylori* via histopathology, 57 (62.6%) of positive *H. pylori* samples were observed in patients with chronic gastritis, 11 (50%) with adenocarcinoma and 31 (44.2%) with superficial gastritis, while only one *H. pylori*-positive out of 5 cases observed in atrophy gastritis patient. ^29^ Histopathology is the first diagnostic method for detection of *H. pylori* and is still widely used as the main diagnostic tool; nevertheless, it has limitations including higher cost, longer turnaround time, and inter-observer variation assessment; experience and skills of the pathologist do matter for the specificity and sensitivity of histopathological diagnosis of *H. pylori,* ^2^ false-positive results can occur due to presence of structures similar to *H. pylori* ^24^ and failure to detect all the positive samples might occur in case of intestinal metaplasia. ^29^ The density and irregular distribution of *H. pylori* can vary at different sites on the gastric mucosa, which might lead to sampling error. ^24^^,^^27^ Moreover, the sensitivity of histology may decrease in patients taking antisecretory therapy, such as, proton pump inhibitor (PPI). ^27^

Molecular tests should be applied as replacements to the traditional method for the identification of *H. pylori*, which are sensitive, rapid, and precise techniques for the specific recognition of *H. pylori* from gastric biopsy specimens and to discover particular mutations related to antimicrobial resistance. ^24^^–^^26^

In this study, identification of *H. pylori* was applied to all biopsies by PCR using specific primers. Specific *H. pylori 16S rRNA* gene is a conserved region of prokaryotic DNA that allows specific identification. However, *H. pylori 16S rRNA* gene's sensitivity and specificity were 46.6% and 78.6%, respectively. The *glmM* gene shows 24.3% sensitivity and 92.5% specificity. In our study, the *ureA* gene showed the lowest sensitivity (23.3%), and 82.3% specificity. Our result aligned with a study conducted by AlNaji *et al.* in 2018, which found that the *glmM* gene is 38.8% lower than the *16S rRNA* gene 95.9%. ^30^ Helaly *et al.* reported similar results (38.5%) for *glmM* gene. ^31^ This low percent of *glmM* ( *ureC*) gene may be due to sequence polymorphism or/in variation to the diversity of strains within the patients that reported in previous studies. ^30^ Also, housekeeping genes are affected by geographical regions and point mutations, Intragenic and recombination are another potential factors. ^32^

The *ureA* gene is a housekeeping gene that is needed for urease enzyme activity. Espinoza *et al.* demonstrated that the amplification of the *ureA* gene was noticed in (86.36%) which was lower than that of the *glmM* gene (100%). ^17^ Smith *et al.* reported that *ureA* gene PCR had a very poor specificity and sensitivity. ^33^ The possible reasons for poor sensitivity of *ureA* and *ureC* ( *glmM*) genes for the detection of *H. pylori* may be that both of them are single-step PCR and thus unable to identify the lower number of bacteria or they were unable to counteract PCR inhibitors in the clinical specimens. ^34^

The *16S rRNA* gene is a useful and commonly used for the primary finding of *H. pylori* use Hp1, Hp2 primers with sensitivity up to 100%. ^30^ Sugimoto *et al.* and Farhadkhani *et al.* reported that the detection of *H. pylori 16S rRNA* gene was greater than the *ureA* gene. They determined that the difference could be due to discrepancy in the primer specificity and sensitivity. Using of *16S rRNA* gene for the detection of *H. pylori* might be more sensitive but could not be as specific as *ureA* gene. ^35^^,^^36^ The poor specificity may be explained by sequence conservation across the bacterial genera and also by possible amplification of nonspecifically human DNA. ^34^ Yet, no 100% specificity or sensitivity for primer sets amplifies *H. pylori ureA* and *16SrRNA* genes. ^35^^,^^36^

## Conclusions

Many tests already exist in the world for diagnosis of *H. pylori* infections. The study results suggest that *H. pylori 16S rRNA* gene detection by the PCR method could be used to diagnose *H. pylori* infections. To avoid false-positive results and increase specificity, we recommend using two conserved target genes to detect *H. pylori* infections.

## Data availability

### Underlying data

Figshare: Underlying data for ‘Comparison of invasive histological and molecular methods in the diagnosis of *Helicobacter pylori* from gastric biopsies of Sudanese patients: a cross-sectional study’.

The project contains the following underlying data:

- Raw data collected from patients with gastritis symptoms: https://doi.org/10.6084/m9.figshare.17072012.v2. ^37^
- Raw gel electrophoresis images: [PCR amplification of *H. pylori* on agarose gel electrophoresis 1.5%]: https://doi.org/10.6084/m9.figshare.18482015.v1. ^38^

Data are available under the terms of the Creative Commons Attribution 4.0 International license (CC-BY 4.0).
