# Supplementary material for: Comparison of invasive histological and molecular methods in the diagnosis of Helicobacter pylori from gastric biopsies of Sudanese patients: a cross-sectional study
Source: F1000Res. 2022 Jun 8;11:113. Originally published 2022 Jan 28. [Version 2] doi: 10.12688/f1000research.75873.2 (PMC9214269; doi:10.12688/f1000research.75873.2)
Supplement: Peer review history

# Peer review: Reviewer response for version 2

**DOI:** 10.5256/f1000research.134815.r140058

**Published:** 2022-06-21

**Authors:** Maxime Pichon

**Affiliations:** 1 Infectious Agents Department, CHU Poitiers, Poitiers, France; 2 INSERM U1070 Pharmacology of Antimicrobial Agents and Antibiotic Resistance, Poitiers, France

The manuscript has been appropriately revised.

Is the work clearly and accurately presented and does it cite the current literature?

Partly

If applicable, is the statistical analysis and its interpretation appropriate?

Yes

Are all the source data underlying the results available to ensure full reproducibility?

Yes

Is the study design appropriate and is the work technically sound?

Partly

Are the conclusions drawn adequately supported by the results?

Partly

Are sufficient details of methods and analysis provided to allow replication by others?

Partly

Reviewer Expertise:

Medical microbiology

I confirm that I have read this submission and believe that I have an appropriate level of expertise to confirm that it is of an acceptable scientific standard.

# Peer review: Reviewer response for version 2

**DOI:** 10.5256/f1000research.134815.r140059

**Published:** 2022-06-10

**Authors:** Stella Ifeanyi Smith

**Affiliations:** 1 Department of Molecular Biology and Biotechnology, Nigeria Institute of Medical Research, Lagos, Nigeria

I have gone through the manuscript and noted the changes made following my query. However, I still noted a few areas that need minor corrections, e.g.:

- Under the introduction, fourth line from the bottom of the first paragraph: should the word not be 'relies' and not 'relays' as written in the manuscript?
- Still under introduction, third paragraph, line 1: polymerase chain reaction, i.e. the c and r should start with small letters.
- Still under the third paragraph, line 2 statement: should be 'PCR is' and not 'PCR which is'.

These are minor corrections which does not affect the decision, which is approved.

Is the work clearly and accurately presented and does it cite the current literature?

Partly

If applicable, is the statistical analysis and its interpretation appropriate?

Yes

Are all the source data underlying the results available to ensure full reproducibility?

Yes

Is the study design appropriate and is the work technically sound?

Partly

Are the conclusions drawn adequately supported by the results?

Partly

Are sufficient details of methods and analysis provided to allow replication by others?

Partly

Reviewer Expertise:

Molecular epidemiology of infectious diseases

I confirm that I have read this submission and believe that I have an appropriate level of expertise to confirm that it is of an acceptable scientific standard.

# Peer review: Reviewer response for version 1

**DOI:** 10.5256/f1000research.79802.r136292

**Published:** 2022-05-25

**Authors:** Stella Ifeanyi Smith

**Affiliations:** 1 Department of Molecular Biology and Biotechnology, Nigeria Institute of Medical Research, Lagos, Nigeria

In the Introduction section, the authors have described invasive histological methods of diagnosis and compared them with basic molecular biology methods and did not include other molecular methods that have been used for *H. pylori* diagnosis and simultaneous detection of resistance including the Fluorescence in situ Hybridization (FISH). These other molecular methods should be included in this section.

 Under the results table, it is expected to see the age of patients by groups, e.g. 1–10 years, and that immediately brings out the age most susceptible to *H. pylori* infection. I also want to believe still under Table 1, that the two hospitals were randomly chosen. The socio-demographic and clinical data are too scanty, kindly give more information.

*Helicobacter pylori* should be italicized and some *H. pylori* have the species name starting with a capital letter, so the species name should start with small letters, not capital letters as the authors have written in some.

 In the conclusion, the authors have written 'urgent need…', there are several available methods now which can be utilized to suit the particular country of origin depending on costs and accuracy. It would have been interesting to see the molecular method compared with both histology and or stool antigen test at the least since histology is not as specific as stool antigen test or urea breath test (the latter might be out of reach due to high cost).

 I, therefore, approve with reservations after my suggestions above have been incorporated into the manuscript.

Is the work clearly and accurately presented and does it cite the current literature?

Partly

If applicable, is the statistical analysis and its interpretation appropriate?

Yes

Are all the source data underlying the results available to ensure full reproducibility?

Yes

Is the study design appropriate and is the work technically sound?

Partly

Are the conclusions drawn adequately supported by the results?

Partly

Are sufficient details of methods and analysis provided to allow replication by others?

Partly

Reviewer Expertise:

Molecular epidemiology of infectious diseases including Helicobacter pylori

I confirm that I have read this submission and believe that I have an appropriate level of expertise to confirm that it is of an acceptable scientific standard, however I have significant reservations, as outlined above.

# Peer review: Reviewer response for version 1

**DOI:** 10.5256/f1000research.79802.r121551

**Published:** 2022-02-11

**Authors:** Maxime Pichon

**Affiliations:** 1 Infectious Agents Department, CHU Poitiers, Poitiers, France; 2 INSERM U1070 Pharmacology of Antimicrobial Agents and Antibiotic Resistance, Poitiers, France

The manuscript by Maram Elnosh *et al. *describes interesting data on the comparison between histology and molecular biology for the detection of *Helicobacter pylori* in gastric biopsies. If the publication is suitable for the editor, modifications remain to be made before indexing.

**Introduction: **

- The introduction needs to be completed, adding information about testing another sample, non-invasive such as stool - for example, see Pichon *et al.*, 2020 ^1^ which demonstrates that invasive sampling is not the sole solution in recent times, especially searching for resistance to primary-line antibiotic resistance. In addition, this reference could provide information on the detection of CLA resistance, not described in this study without good reason.

**Methods:**

- The authors should justify the number of patients they included and their inclusion period.
- Because the authors use a very homemade extraction and PCR process, they must use positive and negative controls. Please describe the results obtained.
- Specify the manufacturers' information for the DNA ladder.

**Results:**

- Table 1: Specify if age is min-max or IQR.
- Table 1: Specify the impact of the two different locations.
- Italicize all gene names.
- The last sentence in the results section of the manuscript should be reworded. The sentence could lead the reader to misunderstand the results. Moreover, the authors indicate a p-value equal to 0 that is statistically impossible, so they have to limit their conclusion to p-value < threshold.
    
- Evaluation of the concordance between the different PCRs tested in this manuscript would be interesting (to be calculated and discussed).
- Table 2: Prefer likelihood ratio instead of PPV and NPV as prevalence was specific.

**Conclusion:**

- Rephrase the first sentence, as many tests already exist in the world there is no urgency.

Is the work clearly and accurately presented and does it cite the current literature?

Partly

If applicable, is the statistical analysis and its interpretation appropriate?

Yes

Are all the source data underlying the results available to ensure full reproducibility?

Yes

Is the study design appropriate and is the work technically sound?

Partly

Are the conclusions drawn adequately supported by the results?

Partly

Are sufficient details of methods and analysis provided to allow replication by others?

Partly

Reviewer Expertise:

Medical microbiology

I confirm that I have read this submission and believe that I have an appropriate level of expertise to confirm that it is of an acceptable scientific standard, however I have significant reservations, as outlined above.

## References

[1] : Diagnostic Accuracy of a Noninvasive Test for Detection of Helicobacter pylori and Resistance to Clarithromycin in Stool by the Amplidiag H. pylori+ClariR Real-Time PCR Assay. *Journal of Clinical Microbiology* .2020;58(4) : 10.1128/JCM.01787-19 10.1128/JCM.01787-19 PMC709874031996442
